# Predicting diagnostic coding in hospitals: individual level effects of price incentives

**DOI:** 10.1007/s10754-021-09314-5

**Published:** 2021-10-06

**Authors:** Kjartan Sarheim Anthun

**Affiliations:** 1 Department of Health Research, SINTEF Digital, Trondheim, Norway; 2 Department of Public Health and Nursing, Norwegian University of Science and Technology, Trondheim, Norway

**Keywords:** Diagnostic coding, Diagnostic related groups, Prospective payment system, Logit regression, Difference in difference

## Abstract

The purpose of this paper is to test if implicit price incentives influence the diagnostic coding of hospital discharges. We estimate if the probability of being coded as a complicated patient was related to a specific price incentive. This paper tests empirically if upcoding can be linked to shifts in patient composition through proxy measures such as age composition, length of stay, readmission rates, mortality- and morbidity of patients. Data about inpatient episodes in Norway in all specialized hospitals in the years 1999–2012 were collected, N = 11 065 330. We examined incentives present in part of the hospital funding system. First, we analyse trends in the proxy measures of diagnostic upcoding: can hospital behavioural changes be seen over time with regards to age composition, readmission rates, length of stay, comorbidity and mortality? Secondly, we examine specific patient groups to see if variations in the price incentive are related to probability of being coded as complicated. In the first years (1999–2003) there was an observed increase in the share of episodes coded as complicated, while the level has become more stable in the years 2004–2012. The analysis showed some indications of upcoding. However, we found no evidence of widespread upcoding fuelled by implicit price incentive, as other issues such as patient characteristics seem to be more important than the price differences. This study adds to previous research by testing individual level predictions. The added value of such analysis is to have better case mix control. We observe the presence of price effects even at individual level.

**Supplementary Information:**

The online version contains supplementary material available at 10.1007/s10754-021-09314-5.

## Introduction

Financing systems come in various forms and shapes, all adapted differently to different contexts and health systems (Jegers et al., 2002). While these financing systems often have many advantages, they may also lead to several adverse effects both related to the quality of care and to how the care is registered. Neby et. al. mentions many different forms of manipulations: selecting, dumping, creaming, skimping, skimming, undertreatment, revolving-door effect, upcoding, overcoding, case-splitting (Neby et al., 2015). In this paper we focus on one specific type of manipulation: upcoding. Upcoding is the wrongful addition of more diagnosis to receive higher reimbursement. This paper examines upcoding at the individual level by utilizing registry data from Norway.

The central government in Norway owns and funds four regional health authorities (RHAs) which are responsible for providing universal access to all specialized care in their regions. These RHAs owns and funds health trusts that typically will consist of one hospital, but some have up to five hospitals. 90 percent of all hospitals are publicly funded and operated (WHO et al., 2020). The remaining 10 percent are private hospitals that also are largely publicly funded, including not-for-profit hospitals with average 96% public funding through long-term contracts with the regional health authorities (WHO et al., 2020). In 1997 the hospitals were owned by the Norwegian counties which were financed by a mix of some small local taxes, and larger national taxes. This led to a budget gaming situation with large deficits, often bailed out by the central government. In 2002 ownership was transferred to the national level with regional health authorities responsible for all secondary services (Hagen & Kaarbøe, 2006). The ownership reform did not alter the incentive structure.

Funding of somatic hospitals in Norway has two main components: one part block grants and one part activity-based funding (ABF). The block grants are criteria-based risk adjusted capitation. The criteria that determine the risk adjustment are age composition, mortality and three work criteria related to individual's health (work assessment allowance, sickness absence and unemployment). The calculation of the risk adjusted capitation is updated each year to reflect the changes in population size of each hospital catchment area and any change in the other criteria.

The second part of the hospital funding system is the main concern for our paper. The activity-based funding does vary according to the kind of, and amount of activity hospitals perform. To reimburse hospitals a system of diagnostic related group (DRG) is utilized. According to each patient's diagnoses, procedures and age, each patient is grouped within a DRG, and each DRG is associated with a specific price. The price for each treatment varies vastly, from quick consultations with a low price to lengthy transplantations that has a high reimbursement. The price is determined by the historic average cost associated with treatment within each DRG. The total share of somatic hospital budgets funded by DRGs have varied between 30 and 60 percent DRG since the introduction of this system in 1997. In 1999–2001 half of the budget was allocated based on ABF. In 2002 and 2004 the ABF share was 60 per cent, while in 2003 and 2005–2012 the share was 40 per cent. We will operationalize the price incentive as average for each DRG and thus do not examine variation over time for each group. Earlier studies that have separated the within and between-effects of prices have found much larger effects in between-variation (Anthun et al., 2017). All public hospitals are funded in the same way. e-based funding from the central government to the hospital trust, and among the RHAs there exists only very slight variations of the risk adjusted capitation scheme.

Various health systems will respond differently to financing systems. Steinbusch et.al. finds that the combination of for-profit hospitals and the use of secondary diagnoses for reimbursement may lead to health systems more prone to upcoding (Steinbusch et al., 2007). Under public systems, there should not really be incentives for abusing the financing system as the hospitals often have the same owner. However, by pressing the system the hospitals might yield marginal budgets improvements.

Earlier research on upcoding has either been on aggregate levels, within specific DRGs, or focused on one-off exogenous shifts in prices (Anthun et al., 2017; Barros & Braun, 2016; Dafny, 2005; Milcent, 2021; Silverman & Skinner, 2004). Our main hypothesis in this paper follows common economic theory that incentives do work. If the hospitals face to seemingly identical groups or coding choices, the hospital will choose the group that yields the largest profit. The larger the incentive, the larger the potential behaviour change. This paper adds to the literature by predicting at individual level, based on complete dataset of all inpatient admissions, and covering a 14-year time span. We do two different approaches for our analysis. First, we analyse trends in proxy measures of diagnostic upcoding: can hospital behavioural changes be seen over time with regards to age composition, readmission rates, length of stay, comorbidity, and mortality? Since we cannot differentiate with certainty the true upcoded from the false upcoded patients we must rely on indirect measures. Methods are descriptive analyses and difference in difference regressions to test if those hospital episodes explicitly exposed to a price incentive are more upcoded. Our final approach is to examine only those patients grouped in DRG pairs to see if variations in the price incentive are related to probability of being coded as complicated.

## Methods and material

### Data

Data on hospital episodes were provided by the Norwegian Patient Register.^1^ The data contained information on the patient (age, sex, ICD-10 diagnoses, and procedures) as well as administrative information (length of stay, type of treatment, DRG codes). To ensure identical definitions of complicated and uncomplicated groups throughout the period, the data was regrouped with one fixed version of the DRG grouper software.^2^ I.e. instead of annual definitions of coding rules, we applied the same rule to all years. Only episodes in inpatient DRGs were selected for inclusion in this paper, N = 11 065 330. Outpatient surgery or day care treatment was not included in the study due to many changes in the funding for such treatments in the included years of this study, and the structure of DRGs for these treatments is not suitable for detecting upcoding.

All episodes were classified as either treatment or control. The treatment group consists of all patients classified in *DRG pairs*. *DRG pairs* are two DRGs linked together as a complicated and uncomplicated couple. The addition of some specific secondary diagnosis and procedures will determine if a patient belongs to one or the other. The complicated patients will yield a higher reimbursement. There are many specific diagnosis and procedures that will "bump" a patient from uncomplicated to complicated, usually some form of relevant comorbidity or more intense hospital treatment. Those patients *not* classified in DRG pairs will be considered as *control* group. To use terminology from difference in difference methodology, the *treatment* the patients (or rather hospitals) are exposed to is the price incentive related to DRG pairs. In analyses in “Trends” section we operationalize this price incentive as the actual added reimbursement weight the hospital may receive if the patient is upcoded from an uncomplicated to a complicated DRG. The patient will be in either control or treatment group by virtue of their main diagnosis.

### Methods and analyses

#### Trends

We apply different methods to test upcoding. Firstly, we examine the trends to ascertain that the development is different between those DRGs that have an implicit incentive for upcoding, compared to “normal” DRGs. Secondly, we utilize the individual level data to predict the probability of each patient being coded as complicated. This section describes further the analysis we perform and discuss our hypothesis for the results.

Our first approach is to do trend comparisons and difference in different (DiD) tests to see if there is a diverging trend between “normal” DRGs, and those patients/DRGs that are exposed to an upcoding incentive in DRG pairs. We do this by examining the trends for (1) age composition; (2) comorbidity, (3) length of stay, (4) readmission rates, and (5) mortality, and perform difference-in-difference analysis for these five indicators. However, we cannot be certain that the assumptions for the difference in difference approach holds. The effect of time is not constant over time, the groups might differ over time, and we cannot ascertain a priori that the treatment and the control groups should have parallel trends. Also, the baseline period is pragmatically chosen as the first year with comparable data, and not the last year before a policy reform. We estimate the development within four groups of DRGs; (1) all DRGs in control group, (2) all DRGs in treatment group (i.e. all DRG pairs), (3) subset of the treatment group: all complicated DRGs, (4) subset of the treatment groups: all uncomplicated DRGs. We first examine graphically the developments in the five indicators for these four categories, year by year.

In the difference in difference analysis, we examine how the trends from 1999 to 2012 develop with regards to age composition, comorbidity, and length of stay, and compare the development from 2008 to 2012 with regards to readmission rates and mortality. The latter two are only available and reliable from 2008 and we cannot use earlier reference years than that. Unfortunately, no earlier baseline makes sense due to different version of the International Classification of Diseases (ICD) in the years preceding 1999.

**Fig. 1:**
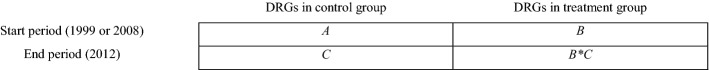
Difference-in-difference setup

We follow a simple difference-in-difference approach by defining a dummy for time (*C*), in this case the last of the years included in each regression (2012). We define a treatment dummy (*B*) for those patients grouped into a DRG pair. Finally, we create an interaction of these two dummies (B*C), and this is the main variable of interest. This interaction will be non-zero if the treatment group has a different trend for the time-period examined than the control group. Figure 1 below illustrates the combination of dummies in the difference-in-difference setup, and Eq. 1 presents the regression equation.

From Eq. 1 we can see that *a* will be a constant in our regression equation. ${x}_{n}$ are other control variables and patient characteristics such as sex, type of DRG (medical or surgical), emergency admittance status. The other outcome variables are included in the control variable vector. We perform one regression analysis for each of the five dependant variables.

$$y=a+{B*\beta }_{0}+C*{\beta }_{1}+{BC*\beta }_{2}+{\beta }_{n}*{x}_{n}+\varepsilon$$

#### Equation 1 Regression equation for difference in difference estimations

Bertrand et.al. suggests that DiD-models often are intrinsically serially correlated, especially when having more than two time points, and among other things they suggest clustering the standard errors if examining groups (Bertrand et al., 2002). We solve this by using only a very simple two time point estimation with the first and the last year in the data, as this should not inflate the serial correlation and our approach will thus yield better standard errors.

While we acknowledge the many forms of hospital behaviour induced by the financing systems (Neby et al., 2015), for the purpose of this paper we focus on and define upcoding as the wrongful coding of uncomplicated patients as complicated. Upcoding is done by the addition of one or more secondary diagnosis that is not relevant for the specific treatment, causing the episode to be grouped in a different group with higher marginal revenue.

#### Trends: assumptions and hypotheses

We assume, a priori, that the patients wrongfully upcoded are less medically complex, and thus receive less costly treatment than the rightfully complicated patients. The true complicated patients are older than their true uncomplicated counterparts. We assume thus that the wrongfully upcoded patients are also on average younger. The upcoding of patients with a lower age will cause the average age of both uncomplicated and complicated groups to be lowered compared to the age composition of DRGs not in DRG pairs. Similar results can be hypothesized with regards to length of stay, readmission rates, comorbidity, and mortality. Under the assumption of either no difference between control and treatment group, or no difference in trend between control and treatment group we hypothesize:

- H_0_: No difference in trends between treatment and control group. This indicates that no upcoding takes place.

If H0 is confirmed, we have an indication that no upcoding takes place. If H0 is falsified, we may have indication of upcoding, however upcoding may not be the only reason for the falsification of H0. We hypothesize further:

- H_1_: If upcoding takes place we assume that severity will be lowered for the treatment group.
- H_2_: If upcoding takes place we expect lowered severity for both complicated and uncomplicated groups.

Our measures of severity by and large positively correlated, so we expect similar direction of results for age composition, length of stay, readmission rates, comorbidity, and mortality. All hypotheses will be tested by visual inspection first, and then H_0_ and H_1_ in differences-in-difference analyses. In these analyses, a negative coefficient for the DD-estimate will be the criteria which we look for. In difference in difference we have four categories: (1) the control group in the reference year, (2) the control group in the year 2012, 3) the treatment group in the reference year, and finally (4) the treatment group in the year 2012. The control group in the reference year is the estimate of the constant, and the reference point for the other estimates. The estimate of year 2012 is interpretable as the coefficient for the control group in the year 2012, while the treatment dummy gives the estimate for the treatment group in the reference year as compared to the control group in the reference year. The difference-in-difference estimate is an interaction term as a product of the time dummy and treatment dummy, and it estimates the difference in the effects of the development for the treatment and the control group (see Fig. 1 above). As a sensitivity analysis, regressions using different years as end year was performed. The results (see tables in appendix) for the years closest to 2012 did not vary much with respect to the main coefficients of concern here, i.e. of the same direction and order of magnitude.

#### The probability of being coded complicated

Our final question is how variations in the price incentive affect the probability of being upcoded? To shed light upon this we analyse further the discharges in the treatment group, i.e. only patients grouped in DRG pairs, N = 5 538 237. Recall that DRG pairs are two diagnosis related groups of the same disease/diagnosis where the complicated patients/treatments are in one group, and the uncomplicated patients/treatments are in another. Our dependant variable is the complicated status of each patient; is the patient grouped in a complicated or an uncomplicated group. Independent variables are the patient’s age, sex, length of stay (logged), case-mix (the DRG weight of the uncomplicated group), the treatment type (emergency/elective, surgical/medical, and major disease categories), regional hospital trust dummies, time trend and finally the price incentive. The latter is the main independent variable of interest. It is measured at the DRG pair level as the price difference between complicated and uncomplicated groups (measured in DRG weights/points). For simplicity we average the price incentive for each DRG. Anthun, Bjørngaard (Anthun et al., 2017) shows variations within DRGs but found much smaller effects than between groups. We perform logit estimations with dependent variable being complicated status of episode.

We did a separate analysis of 2008–2012 data to control for out of hospital mortality. Date of death for the deceased was collected from the National Registry and linked in the data by Norwegian Patient Register. We measured time to death as number of days to death after admission, and for the regression we created a dummy with value 1 for those that died within 30 days, and 0 for those that did not. Other operationalisations could have been used, however, to avoid systematic bias in the 2012 data we wanted to keep the measure of time to death as short as possible. Due to the data creation process in 2013/2014 not all possible deaths had yet been linked, and this led to a lowered mortality rate in 2012 than other years.

#### The probability of being coded complicated: assumptions and hypotheses

With regards to those patients coded up in a different group we expect all common issues related to case-mix to be important for the prediction of complicated status. Our analysis controls for age, gender, length of stay and comorbidity, and our aim is to test if price incentive at the individual level has an impact on the probability after controlling for these. If wrongfully upcoding takes place, we expect price to be an important factor and thus positively correlated. If no wrongfully upcoding takes place, we expect not to find any effect of price at all.

- H_0_: There is no impact from price incentive on the probability.
- H_1_: There is positive association from price incentive to the probability of being coded complicated.

H_0_ will be if the coefficient of the price incentive is 0 (or 1 as odds-ratio), and H_1_ will be true if the coefficient is > 0 (or odds ratio > 1).

## Results

The main purpose of this paper is to analyse if upcoding takes place, and if so how upcoding is related to price. Our main interest is explaining the development of complicated cases in the DRG pairs (see Table 1). In 1999 only 27.9 per cent was complicated. This increased steeply for the first few years before reaching a maximum in 2006 at 44.0 per cent and after that declined slightly to 42.5 in 2012.

**Table 1:** Percentage of cases in DRG pairs classified as complicated

Year	1999	2000	2001	2002	2003	2004	2005	2006	2007	2008	2009	2010	2011	2012
% Compl	27.9	31.0	34.1	39.9	42.0	42.4	43.3	44.0	43.3	43.7	42.8	42.6	42.6	42.5

We will first discuss trends for the possible related measures or outcomes due to upcoding: indirect shifts in proxy measures. We examine mean age, length of stay, readmission rates, comorbidity, and mortality. Further we do differences-in-difference regressions, before testing the probability of being coded as complicated.

### Trends

**Fig. 2:**
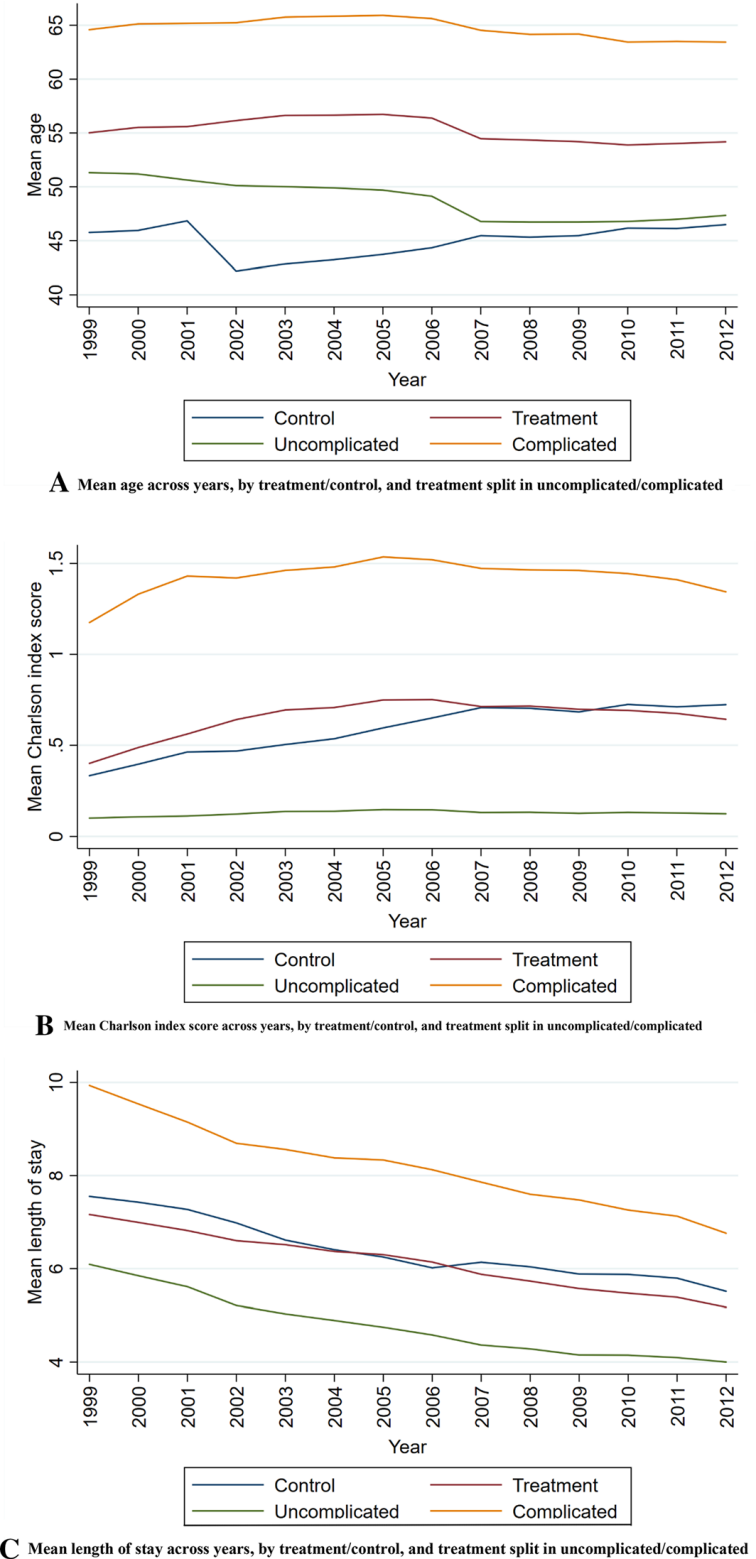
**A** Mean age across years, by treatment/control, and treatment split in uncomplicated/complicated. **B** Mean Charlson index score across years, by treatment/control, and treatment split in uncomplicated/complicated. **C** Mean length of stay across years, by treatment/control, and treatment split in uncomplicated/complicated

The average age of patients in the control group was 45.8 in 1999 and 55.0 in the treatment group, see Fig. 2a and table 5 in the online appendix. There was an age difference of 9.3 years in 1999 which increased to 13.9 in 2002. This difference drops to 7.7 years in 2012 because of an increase in the control group after 2002, and the treatment group declines after 2005. If we split treatment in complicated and uncomplicated, we observe a falling trend for uncomplicated, while the complicated groups increase marginally from 1999 to 2005 and then declines somewhat.

Comorbidity is important when it comes to upcoding since the real complicated patients are likely to have more comorbidities. Comorbidity is closely related to the actual measure of upcoding, so it might be difficult to untangle the two concepts. We have calculated the Charlson index (Charlson et al., 1987) for each episode. Charlson is a common comorbidity measure that has been thoroughly measured and validated earlier and shown to be a good predictor of mortality. The index is an additive weighted index in which different comorbidities (registered as secondary diagnoses) add up to an increasing measure of comorbidity, spanning from 0 to 16. Figure 2b shows the aggregated development of the index in the period 1999–2012. The patients in the treatment group have the value 0.40 in 1999 while the average for the control patients was 0.33. There was a diverging trend for treatment group and control group in the first 5 years, before they converged to being equal in 2007–2009, and after that the treatment marginally declines and the control marginally increases. For the uncomplicated groups the index was, not surprisingly, very low and stable from 1999 to 2012. The complicated group has much higher values, increasing from 1.18 in 1999 to 1.54 in 2005—after follows a slow decline to 1.34 in 2012. Over the entire period, the growth in the control group was higher than the growth of the treatment group.

In the period the length of stay declined for both the control group and treatment group, as seen in Fig. 2c. This is by and large due to improved technology, but also the financing system might cause some further shifts towards smaller length of stay. Regarding income, it will be more beneficial for the hospital to treat one patient twice for one week, than the same patient continuous for two weeks. The control group has on average 7.6 inpatient days in 1999 decreasing to 5.5 in 2012. The treatment group has a lower length of stay with 7.2 in 1999 decreasing to 5.2 in 2012. There are some changes in the distance between the treatment and control group, but the difference is around 0.4 in most years. If we also examine the two subgroups in the treatment group, we see that the uncomplicated cases on average have 6.1 inpatient days in 1999, decreasing to 4.0 in 2012. The complicated cases start at 9.9 in in 1999 and ends up at 6.8 in 2012. One could argue that it is easier to reduce length of stay where it was longest in the beginning; however, we see equal numbers for the control, treatment, and uncomplicated groups.

**Fig. 3:**
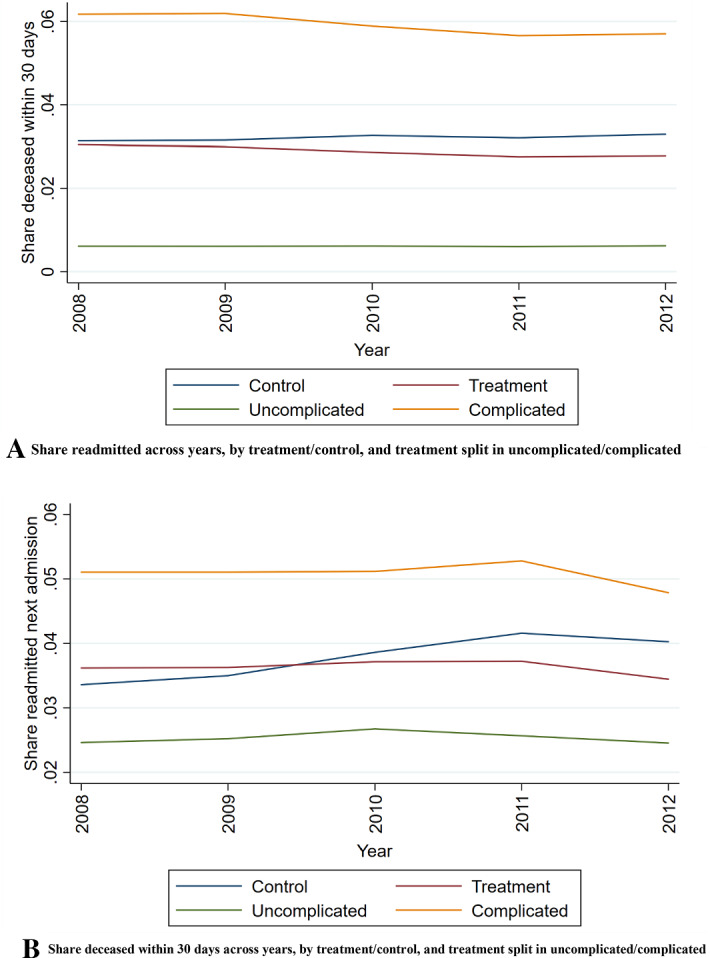
**A** Share readmitted across years, by treatment/control, and treatment split in uncomplicated/complicated. **B** Share deceased within 30 days across years, by treatment/control, and treatment split in uncomplicated/complicated

For readmissions (Fig. 3a) and 30-day mortality (Fig. 3b) the time-series is shorter, showing negative development for the treatment groups and a positive for the control group. The mortality is 0.031 in the control group in 2008, and 0.030 in the treatment groups. The trends diverge in the period, as the mortality of the complicated cases of the treatment group decreases from 0.062 in 2008 to 0.057 in 2012. Readmissions shows and increasing trend for control group patients and decreasing for the treatment group patients.

### Covariates of the trends in proxy measures of upcoding

In this section we continue the trend analyses by setting up difference in difference regression analyses for the five proxy measures of upcoding. Age, comorbidity, and length of stay are continuous variables and will be tested by OLS regression while readmission and mortality are dichotomous and will be tested with logistic regression. The five outcome measures are correlated, and in the following regressions we have controlled for these where possible since we want to be sure that for instance age is not confounding readmissions rates. The results will be more reliable if we find interaction effects of time and treatment group (i.e. the Difference-in-Difference estimate) that cannot be explained by the other dependant variables and not just the independent variables.

In the regression for age, we find a negative interaction (i.e. the difference in difference estimate) after controlling for the other variables. The time effect is 3.87 and the treatment group effect is 4.52, while the difference in difference estimate is -3.02. That implies that for age, the trend is negative for the treatment group, but positive for the control group.

For comorbidity, as measured by the Charlson index, the estimates are different than for age. In the trend analyses (Fig. 2a–c above) the results indicated that there was upcoding as we saw the control group had a larger increase than the treatment group. After controlling for other issues in the regression, we see in Table 2 that the interaction effect is positive although small at 0.02.

**Table 2:** Regression coefficients for difference-in-difference regressions, coefficients and 95% confidence intervals

Variables	1: Age	2: Charlson comorbidity index	3: Length of stay	4: Readmission	5: Mortality within 30 days of admission
Year 2012	3.87***	0.30***	− 2.79***	1.11***	1.03**
	(3.76–3.97)	(0.29–0.31)	(− 2.83–2.75)	(1.08–1.13)	(1.00–1.06)
Treatment group	4.52***	− 0.10***	− 0.73***	0.93***	0.72***
	(4.41–4.63)	(− 0.11–0.09)	(− 0.77–0.68)	(0.91–0.96)	(0.70–0.74)
Interaction (DD estimate)	− 3.02***	0.02***	0.59***	0.86***	0.92***
	(− 3.16–2.88)	(0.01–0.03)	(0.54–0.65)	(0.84–0.89)	(0.89–0.96)
Sex (female)	3.01***	− 0.12***	− 0.06***	0.74***	0.87***
	(2.94–3.09)	(− 0.12–0.12)	(− 0.09–0.03)	(0.73–0.75)	(0.85–0.88)
Medical DRG	0.27***	0.28***	− 2.08***	0.32***	0.97*
	(0.18–0.36)	(0.27–0.28)	(− 2.12–2.04)	(0.32–0.33)	(0.94–1.00)
Emergency admittance	1.95***	− 0.08***	0.64***	1.53***	4.21***
	(1.87–2.04)	(− 0.09–0.08)	(0.61–0.67)	(1.50–1.56)	(4.07–4.35)
Age (years)		0.01***	0.05***	1.00***	1.05***
		(0.01–0.01)	(0.05–0.05)	(1.00–1.00)	(1.05–1.06)
Weighted Charlson Sum	3.10***		0.61***	1.10***	1.36***
	(3.08–3.13)		(0.60–0.62)	(1.09–1.10)	(1.35–1.36)
Length of stay	0.32***	0.01***		1.01***	0.99***
	(0.31–0.32)	(0.01–0.01)		(1.01–1.01)	(0.99–0.99)
Patient dead within 30 days of admittance				0.86***	
				(0.83–0.90)	
Patient readmitted in next hospital episode					1.11***
					(1.06–1.16)
Constant	42.02***	− 0.51***	7.29***	0.11***	0.00***
	(41.81–42.22)	(− 0.52–0.50)	(7.21–7.37)	(0.10–0.11)	(0.00–0.00)
Observations	1,519,431	1,519,431	1,519,431	1,678,667	1,678,667
R-squared	0.38	0.16	0.09	0.12	0.25
Reference year	1999	1999	1999	2008	2008
Type of estimate/coefficient	OLS Coefficient	OLS Coefficient	OLSCoefficient	Logistic Odds ratio	Logistic Odds ratio

95% confidence interval in parentheses. R-squared is pseudo R-squared for logistic regressions

Dummies for main disease categories (MDC) and month of year are not displayed in table

***p < 0.01, **p < 0.05, *p < 0.1

Length of stay has had a decreasing trend for both treatment and control groups from 1999 to 2012, if we only look at the dummies in our regression, we estimate a decrease from 7.29 by 2.79 to 4.50 for the control group. The treatment group starts at 7.29–0.73 = 6.56 and ends at 7.29–2.79–0.73 + 0.59 at 4.36 in 2012. The differences-in-difference estimate is quite large considering the small discrepancies of the trends found in Fig. 2a–c and table 7. This indicates that the uncontrolled results were confounded by variables that we now have controlled for, possibly medical DRGs as the variable has a large estimate (Table 2).

Readmission and mortality are logistic regressions, where an odds ratio of < 1 indicates a negative association while > 1 is a positive association. For both readmission and mortality, we find positive trend for control group (year 2012 dummy) and negative for the treatment group and negative interaction (DiD estimate). We included all other dependent variables in the regressions^3^ to control for important case mix information. As expected, these are mostly all positively correlated, aside for marginally negative effect from length of stay on mortality, and a negative association of readmission and mortality. The latter is negative since we measure mortality from admittance and not discharge. So, for the deceased there is less days to be readmitted within and thus the association is negative. As for length of stay, the results can imply that longer length of stay reduces mortality marginally.

### Probability of being coded as complicated

We perform logit regressions on the treatment group to analyse how different independent variables co-varies with the complicated grouping outcome. Results are presented in Table 3. We start with a simple model where the price incentive is the only independent variable, the odds ratio is 1.39. We then add, in another model, the patient characteristics and see a slightly lowered odds ratio of 1.20. Adding temporal variables (linear time trend for year and dummies for months to control for seasonal variation) does not alter this much. However, the model including information about the patient’s case-mix (dummy for medical DRGs, (logged) length of stay, dummy for emergency admissions, and DRG weight of uncomplicated group) yielded a better fit of the regression, and a lower estimate of the price incentive with odds ratio of 1.15.

**Table 3:** Logistic regressions of complicated status, odds ratio and 95% confidence intervals

Dependant variable: complicated patient	1: Only price	2: Patient characteristics	3: Temporal	4: Case mix	5: 2008–2012 Only price	6: 2008–2012 Case mix	7: 2008–2012 Readmission and mortality
Price incentive	1.39***	1.20***	1.22***	1.15***	1.64***	1.08***	1.05***
	(1.39–1.40)	(1.20–1.20)	(1.21–1.22)	(1.14–1.15)	(1.63–1.65)	(1.07–1.09)	(1.04–1.06)
Age (years)		1.03***	1.03***	1.03***		1.03***	1.03***
		(1.03–1.03)	(1.03–1.03)	(1.03–1.03)		(1.03–1.03)	(1.03–1.03)
Sex (female)		0.92***	0.91***	0.84***		0.83***	0.84***
		(0.92–0.92)	(0.91–0.92)	(0.84–0.84)		(0.82–0.84)	(0.83–0.84)
Time trend (years since reference year)			1.05***	1.06***		1.00***	1.00***
			(1.05–1.05)	(1.06–1.07)		(1.00–1.01)	(1.00–1.01)
Medical DRG				1.88***		1.58***	1.49***
				(1.86–1.89)		(1.56–1.60)	(1.48–1.51)
LOS (logged)				2.15***		2.54***	2.58***
				(2.14–2.15)		(2.52–2.55)	(2.57–2.59)
Emergency admittance				1.37***		1.33***	1.27***
				(1.37–1.38)		(1.32–1.34)	(1.26–1.28)
DRG weight (of uncomplicated group)				1.33***		1.34***	1.26***
				(1.33–1.34)		(1.33–1.35)	(1.25–1.26)
Patient readmitted in next hospital episode							1.50***
							(1.47–1.52)
Patient dead within 30 days of admittance							4.70***
							(4.58–4.82)
Observations	5,538,034	5,538,034	5,538,034	5,538,034	2,181,053	2,181,053	2,181,053
Pseudo R^2^	0,005	0,088	0,094	0,164	0,010	0,176	0,183

95% Confidence interval in parentheses

In models 3, 4, 6 and 7 dummies for months omitted from table. In models 4 and 6 and 7 main disease categories dummies (MDCs) are omitted from the table

Price incentive defined as the DRG weight increase that would be reimbursed if the patient was grouped in complicated group

***p < 0.01, **p < 0.05, *p < 0.1

We also examined three models with only data from 2008 to 2012. The first is again an empty model with just the price incentive, the second has all the same variables as the case mix model while the final also includes dummy for readmissions and dummy for mortality (if death within 30 days of admittance). Here we see that the effect of price incentive decreases, and the effect is zero for the model including readmissions and mortality (odds ratio is 1).

The patient’s age is an important case mix adjustor.^4^ In all models we find an odds ratio of 1.03. This means that for every additional year of age, a patient is three percentage more at risk of being coded as complicated. Sex also has a strong effect as women are less likely to be coded as complicated. The time trend is strong in the models with the whole data period (all fourteen years), but not so much in the subset from 2008 to 2012. This reflects the levelling out we remember from Table 1. However, there is not a negative coefficient, which suggests that the slightly negative overall development of complicated cases from 2008 to 2012, from 43.7 to 42.5 per cent nationally, is explained by the individual characteristics.

Patients grouped in medical DRGs (as opposed to surgical DRGs) are more likely to be complicated. Similarly, the emergency admissions are more likely than planned/elective admissions to be coded as complicated. Other known case-mix related characteristics have an important and strongly positive association with the probability of being coded as complicated. Length of stay is an important factor here^5^ with a large coefficient in all models. The DRG weight of the uncomplicated is the relative treatment cost of the DRG pair.^6^ We observe that the larger DRG weights have a higher probability of being complicated. This indicates that the DRG pairs that are already more expensive will have a higher complicated share than those DRG pairs which are less expensive. The latter could also be a result related to the size of the groups, as the costlier DRG will be less numerous than the less costly DRGs.

In the final model we tested if including a dummy for those patients readmitted in the next hospital episode, and a dummy for those admissions were the patient deceases within 30 days of admittance. These independent variables have a huge impact of the probability of being coded as complicated, the patients that are dead within 30 days of admittance are more than four times as likely to be complicated patients as opposed to those patients that survive the first 30 days.

## Discussion

Our visual trend analyses found support for indirect traces of upcoding for all related measures of severity we studied. As we remember from the hypotheses, if wrongful upcoding takes place we expect reduced severity. Age, comorbidity, length of stay, readmissions and mortality all showed very similar results. The trend of the patients where the hospital had an incentive for upcoding (treatment group: DRG pairs) had lowered severity trend than that of the control group where there was no incentive for coding up. Also, all these measures showed that the severities of both complicated and uncomplicated groups were lowered. This indicates that potentially some patients have been wrongfully upcoded from uncomplicated to complicated groups. After regressions with controls, we found continued support for this for age, readmissions, and mortality. However, for comorbidity and length of stay the trend was positive for the differences-in-difference estimate after controlling for other patient characteristics.

We found a positive association of price incentive and complicated coding. However, this effect was diminished when controlling for other patient characteristics and remains only very small in the complete model. When only examining the last five years we found no effect. This gives us some support for the hypothesis that there is an association of incentive and coding, but also reason to speculate whether a lot of the potential for upcoding now has been exploited and the share of complicated patients seem to have levelled out at around 42 per cent. It has been argued in the literature on activity-based funding that the presence of such funding schemes will increase the quality and completeness of coding (Fisher et al., 1992; O'Reilly, et al., 2012). In a study on coding of heart failure a similar sign of levelling out was shown (Sacarny, 2018). The Norwegian directorate of Health, the responsible authority on the implementation of the funding scheme, had previously defined DRG creep as "patients being coded as more complete, resulting in an increase in case mix index".^7^

The levelling out occurred after the 2002 ownership reform of the Norwegian hospitals, but it is difficult to assess if the ownership reform that caused this. The trends indicate no abrupt change around this time, rather the slowly levelling out. Since upcoding is theoretically only possible for a part of the hospital activity, and hospital funding in Norway being a null sum game, there are limits to how alluring upcoding is. The Norwegian directorate of Health organizes a *settlement committee [Avregningsutvalg]* that audits the funding scheme and relevant medical coding. The committee yearly reduces the activity-based funding transfers to hospital if they uncover misuse (such as upcoding). The size of reduction is however typically less than 0.1 percent of the total budgets for hospitals. One can only speculate if the presence of such a committee will limit any further wrongful upcoding.

Can these results be classified as further evidence of upcoding? These analyses are at the individual level over a large period, so after controlling for patient characteristics and case mix adjusting, there should not be any effect of the price incentive in a public NHS-style system. However, we must also remember that we have not measured here the real upcoding but rather the upcoding in regrouped data, i.e. not all the “upcoded” are real. So, while the comparability over time is better, a small percentage of those that we have classified as upcoded might in fact be upcoded due to the specific classification rules of 2011 used here, and not due to the yearly incentives that are real for the hospitals.

Another issue is whether the methods we have chosen are suitable for these analyses. Can parallel trends be assumed for age, readmission, and mortality? Ideally, we should have had a period before the DRGs were used for reimbursement purposes as “before the intervention” in the DD-terminology. However, the introduction of the 10th Edition of the ICD in Norway in 1999 acts as a baseline which is difficult to go beyond in history to analyse the use the diagnoses. Nevertheless, the estimations and analyses are a test of how the development in the trend of one group compares to the development in the trend of another group. Irrespective of how the origin and separation of these two groups are, we have demonstrated that their paths are different. Some of these differences can be accounted by (as case-mix), as we have seen in the logit analysis of complicated status that case mix is a very important part of the understanding of the grouping of a patient. Other differences remain, and we have seen how the treatment group has a different development than what could be expected if there was no difference between the incentives for coding amongst treatment and control group.

We have tried to examine as long a period as possible. The present study includes an unprecedented long time series of 14 years. A longer time span of individual level data was not possible to collect at the time of data construction. The share of complicated cases has levelled out, so the effect of incentives seems to be an initial adaption to a new system and not a perpetual creep. The DRG system seems to be well adapted to Norway. But the system is continuously developing by extending and adapting to the hospital reality. Latest development includes rehabilitation, psychiatric care, substance abuse and specific medications.

In recent years a few studies have been published in upcoding in Norway (Anthun et al., 2017; Januleviciute et al., 2016; Melberg et al., 2006). Januleviciute et al. showed that upcoding was present in Norway, but only to a small extent. Melberg et al. also found that hospitals were responsive to price changes, and Anthun et al. demonstrated only small effects within groups (i.e. not high price responsiveness) but larger differences between groups, indicating that case-mix is important. This present study extends these analyses by not testing on aggregate data, but rather individual level predictions of complicated status. In empty models testing only for price incentive we saw that there was a large effect, i.e. that the price had a large impact on the probability of being coded as complicated. However, this turns out to be largely an effect of case-mix, when controlling for case-mix a large part of the price incentive effect is gone. Analysing with individual level data provides important insights, but it does also allow for more noise to enter the models, as the pseudo R^2^ remains quite small. Analysis on individual level data seems to corroborate results from earlier studies, but also shows that a large part of the effect is due to case mix differences in groups. If the presence of an incentive is enough to drive upcoding, why do the hospitals not increase upcoding more? The current level of complicated coding does most likely reflect the actual need of the patients and the treatment they are given. An increase in the need, or an increase in the treatment intensity will likely result in future shifts in coding; but seemingly the potential shift related to this specific incentive has already been exploited.

Luo and Gallagher (2010: 600) claim that “DRG upcoding in a public hospital system is most likely due to unintentional errors such as misspecification by the doctor or misunderstanding by the coder”(Luo & Gallagher, 2010). Steinbusch et al. also points to health system characteristics. Norway is a public hospital system with government ownership of all hospitals. Upcoding can be viewed as a zero-sum game as the overall allocations to the hospital sector should not be altered by any one hospital decisions on upcoding. Hospitals can increase reimbursement by doing this, and there are several known examples of illegal upcoding even in Norway (Neby et al., 2015). However, in our results we find not any one large shift in upcoding at either time or place, rather we have demonstrated a very slow DRG creep over time, across many different DRG groups and across all hospitals. The price incentive effect is present, although very weak. If one considers the period where the most upcoding took place, this was where many hospitals were under economic distress, and where the central government had soft budgets constrains on the hospitals. This can be viewed by the hospitals as a larger opportunity for upcoding, and we see the results of this even on the individual level.

## Supplementary Information

Below is the link to the electronic supplementary material.

Supplementary file1 (DOCX 61 kb)

## Footnotes

1 The interpretation and reporting of these data are the sole responsibility of the author, and no endorsement by the Norwegian Patient Register is intended nor should be inferred.

2 Software (grouper version NOR2011co1F) was supplied by the Finnish firm DataWell Oy based upon classifications by the Nordic CaseMix Centre. The regrouping was done by the author.

3 Since readmissions and mortality only were reliably measured from 2008 to 2012 these were not included in the regressions that had 1999 as a reference year.

4 All models including age were also tested with age squared, however in logit regression one can assume that linear variables will be curve linearly interpreted, and the average complicated coding related to age seems to be linear (see Fig. 4 in appendix). Age squared always contributed significantly to the models, however having millions of cases most variables will become significant. The contribution to pseudo R^2^ was only marginal (< 0.003 increase). To ease the interpretability of regressions we only present results with linear age effect.

5 Length of stay was logged to ensure that the models would converge.

6 The price incentive is the difference in the DRG weight of the complicated and uncomplicated groups.

7 Translated by the author, based on an early definition from Helsedirektoratet. *DRG-ordliste [DRG Dictionary]*. 2011. This definition is no longer published.
