# Supplementary material for: Predicting diagnostic coding in hospitals: individual level effects of price incentives
Source: Int J Health Econ Manag. 2021 Oct 6;22(2):129–46. doi: 10.1007/s10754-021-09314-5 (PMC9090893; doi:10.1007/s10754-021-09314-5)
Supplement: Supplementary file 1 — Supplementary file1 (DOCX 61 kb) [file 10754_2021_9314_MOESM1_ESM.docx]

# APPENDIX

Table 4 Number of cases in control group, treatment group, difference of treatment and control, treatment group split in uncomplicated and complicated cases

| **Year** | **Control group** | **Treatment group** | **Difference treatment and control group** | **Uncomplicated cases** | **Complicated cases** |
| --- | --- | --- | --- | --- | --- |
| 1999 | 332 944 | 342 796 | 9 852 | 247 272 | 95 524 |
| 2000 | 332 450 | 339 542 | 7 092 | 234 176 | 105 366 |
| 2001 | 345 387 | 351 751 | 6 364 | 231 858 | 119 893 |
| 2002 | 393 780 | 357 757 | -36 023 | 214 900 | 142 857 |
| 2003 | 418 633 | 372 389 | -46 244 | 215 918 | 156 471 |
| 2004 | 423 932 | 377 519 | -46 413 | 217 540 | 159 979 |
| 2005 | 433 876 | 386 078 | -47 798 | 218 890 | 167 188 |
| 2006 | 449 620 | 398 123 | -51 497 | 222 915 | 175 208 |
| 2007 | 403 612 | 431 026 | 27 414 | 244 372 | 186 654 |
| 2008 | 403 971 | 431 005 | 27 034 | 242 592 | 188 413 |
| 2009 | 402 875 | 428 936 | 26 061 | 245 517 | 183 419 |
| 2010 | 391 372 | 435 037 | 43 665 | 249 618 | 185 419 |
| 2011 | 392 526 | 440 546 | 48 020 | 252 821 | 187 725 |
| 2012 | 402 115 | 445 732 | 43 617 | 256 478 | 189 254 |

Table 5 Average age in control group, treatment group, difference of treatment and control, treatment group split in uncomplicated and complicated cases

| **Year** | **Control group** | **Treatment group** | **Difference treatment and control group** | **Uncomplicated cases** | **Complicated cases** |
| --- | --- | --- | --- | --- | --- |
| 1999 | 45.76 | 55.02 | 9.26 | 51.32 | 64.59 |
| 2000 | 45.95 | 55.51 | 9.56 | 51.19 | 65.12 |
| 2001 | 46.84 | 55.59 | 8.75 | 50.63 | 65.17 |
| 2002 | 42.20 | 56.15 | 13.95 | 50.11 | 65.23 |
| 2003 | 42.84 | 56.63 | 13.78 | 50.01 | 65.76 |
| 2004 | 43.24 | 56.65 | 13.41 | 49.90 | 65.84 |
| 2005 | 43.73 | 56.72 | 12.99 | 49.69 | 65.92 |
| 2006 | 44.34 | 56.38 | 12.04 | 49.12 | 65.62 |
| 2007 | 45.47 | 54.46 | 9.00 | 46.78 | 64.53 |
| 2008 | 45.32 | 54.34 | 9.02 | 46.73 | 64.15 |
| 2009 | 45.47 | 54.19 | 8.72 | 46.73 | 64.18 |
| 2010 | 46.16 | 53.88 | 7.72 | 46.79 | 63.44 |
| 2011 | 46.12 | 54.02 | 7.90 | 46.98 | 63.50 |
| 2012 | 46.49 | 54.18 | 7.69 | 47.35 | 63.43 |

Table 6 Average Charlson score in control group, treatment group, difference of treatment and control, treatment group split in uncomplicated and complicated cases

| **Year** | **Control group** | **Treatment group** | **Difference treatment and control group** | **Uncomplicated cases** | **Complicated cases** |
| --- | --- | --- | --- | --- | --- |
| 1999 | 0.332 | 0.400 | 0.068 | 0.101 | 1.175 |
| 2000 | 0.396 | 0.487 | 0.092 | 0.108 | 1.331 |
| 2001 | 0.463 | 0.562 | 0.099 | 0.112 | 1.431 |
| 2002 | 0.468 | 0.641 | 0.173 | 0.123 | 1.420 |
| 2003 | 0.504 | 0.694 | 0.190 | 0.137 | 1.462 |
| 2004 | 0.535 | 0.707 | 0.172 | 0.138 | 1.481 |
| 2005 | 0.595 | 0.749 | 0.154 | 0.148 | 1.536 |
| 2006 | 0.650 | 0.751 | 0.101 | 0.146 | 1.521 |
| 2007 | 0.707 | 0.713 | 0.006 | 0.132 | 1.473 |
| 2008 | 0.703 | 0.715 | 0.012 | 0.133 | 1.465 |
| 2009 | 0.683 | 0.698 | 0.014 | 0.127 | 1.462 |
| 2010 | 0.724 | 0.692 | -0.033 | 0.132 | 1.444 |
| 2011 | 0.711 | 0.675 | -0.036 | 0.129 | 1.411 |
| 2012 | 0.723 | 0.642 | -0.081 | 0.125 | 1.344 |

Table 7 Average length of stay in control group, treatment group, difference of treatment and control, treatment group split in uncomplicated and complicated cases

| **Year** | **Control group** | **Treatment group** | **Difference treatment and control group** | **Uncomplicated cases** | **Complicated cases** |
| --- | --- | --- | --- | --- | --- |
| 1999 | 7.55 | 7.16 | -0.39 | 6.09 | 9.94 |
| 2000 | 7.43 | 6.99 | -0.44 | 5.85 | 9.54 |
| 2001 | 7.27 | 6.82 | -0.45 | 5.61 | 9.15 |
| 2002 | 6.98 | 6.60 | -0.38 | 5.21 | 8.69 |
| 2003 | 6.61 | 6.52 | -0.10 | 5.03 | 8.56 |
| 2004 | 6.41 | 6.37 | -0.04 | 4.89 | 8.38 |
| 2005 | 6.25 | 6.30 | 0.05 | 4.75 | 8.34 |
| 2006 | 6.02 | 6.14 | 0.12 | 4.58 | 8.13 |
| 2007 | 6.14 | 5.88 | -0.26 | 4.37 | 7.86 |
| 2008 | 6.04 | 5.73 | -0.31 | 4.28 | 7.60 |
| 2009 | 5.88 | 5.57 | -0.31 | 4.15 | 7.48 |
| 2010 | 5.88 | 5.47 | -0.40 | 4.15 | 7.26 |
| 2011 | 5.80 | 5.39 | -0.41 | 4.10 | 7.13 |
| 2012 | 5.52 | 5.17 | -0.34 | 4.00 | 6.76 |

Table 8 Share of admissions classified as readmissions within 30 days in control group, treatment group, difference of treatment and control, treatment group split in uncomplicated and complicated cases

| **Year** | **Control group** | **Treatment group** | **Difference treatment and control group** | **Uncomplicated cases** | **Complicated cases** |
| --- | --- | --- | --- | --- | --- |
| 2008 | 0.034 | 0.036 | 0.003 | 0.025 | 0.051 |
| 2009 | 0.035 | 0.036 | 0.001 | 0.025 | 0.051 |
| 2010 | 0.039 | 0.037 | -0.001 | 0.027 | 0.051 |
| 2011 | 0.042 | 0.037 | -0.004 | 0.026 | 0.053 |
| 2012 | 0.040 | 0.034 | -0.006 | 0.025 | 0.048 |

Table 9 Share of admissions where patient is deceased within 30 days of admission in control group, treatment group, difference of treatment and control, treatment group split in uncomplicated and complicated cases

| **Year** | **Control group** | **Treatment group** | **Difference treatment and control group** | **Uncomplicated cases** | **Complicated cases** |
| --- | --- | --- | --- | --- | --- |
| 2008 | 0.031 | 0.030 | -0.001 | 0.006 | 0.062 |
| 2009 | 0.032 | 0.030 | -0.002 | 0.006 | 0.062 |
| 2010 | 0.033 | 0.029 | -0.004 | 0.006 | 0.059 |
| 2011 | 0.032 | 0.028 | -0.004 | 0.006 | 0.057 |
| 2012 | 0.033 | 0.028 | -0.005 | 0.006 | 0.057 |


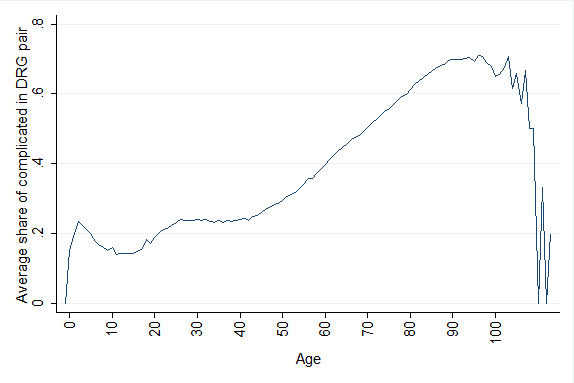


Figure 4 Average share of complicated patients in DRG pairs, by age

Table 10 Coefficients of interest from sensitivity analyses with varying end year in difference-in-difference analysis. Same included controls as table 2. Ordinary least squares.

|  | **Length of stay** | | | | | | | | | | | | |
| --- | --- | --- | --- | --- | --- | --- | --- | --- | --- | --- | --- | --- | --- |
| **VARIABLES** | **Treatment year 2000** | **Treatment year 2001** | **Treatment year 2002** | **Treatment year 2003** | **Treatment year 2004** | **Treatment year 2005** | **Treatment year 2006** | **Treatment year 2007** | **Treatment year 2008** | **Treatment year 2009** | **Treatment year 2010** | **Treatment year 2011** | **Treatment year 2012** |
| Treatment year | -0.30*** | -0.55*** | -0.93*** | -1.27*** | -1.51*** | -1.66*** | -1.82*** | -2.06*** | -2.19*** | -2.38*** | -2.51*** | -2.53*** | -2.79*** |
| Treatment group | -0.55*** | -0.53*** | -0.61*** | -0.66*** | -0.66*** | -0.66*** | -0.65*** | -0.70*** | -0.70*** | -0.70*** | -0.71*** | -0.72*** | -0.73*** |
| Interaction (DD estimate) | 0.00 | 0.03 | 0.10*** | 0.29*** | 0.36*** | 0.40*** | 0.43*** | 0.52*** | 0.52*** | 0.55*** | 0.58*** | 0.53*** | 0.59*** |
| R-squared | 0.09 | 0.09 | 0.10 | 0.09 | 0.09 | 0.09 | 0.10 | 0.10 | 0.10 | 0.10 | 0.09 | 0.10 | 0.09 |
|  | **Age** | | | | | | | | | | | | |
| **VARIABLES** | **Treatment year 2000** | **Treatment year 2001** | **Treatment year 2002** | **Treatment year 2003** | **Treatment year 2004** | **Treatment year 2005** | **Treatment year 2006** | **Treatment year 2007** | **Treatment year 2008** | **Treatment year 2009** | **Treatment year 2010** | **Treatment year 2011** | **Treatment year 2012** |
| Treatment year | 0.32*** | 0.87*** | 1.31*** | 1.92*** | 2.25*** | 2.52*** | 2.74*** | 3.42*** | 3.50*** | 4.04*** | 3.81*** | 3.91*** | 3.87*** |
| Treatment group | 3.85*** | 3.92*** | 4.07*** | 4.01*** | 4.15*** | 4.22*** | 4.26*** | 4.48*** | 4.57*** | 4.55*** | 4.53*** | 4.65*** | 4.52*** |
| Interaction (DD estimate) | -0.22*** | -1.07*** | -1.21*** | -1.60*** | -1.88*** | -2.11*** | -2.38*** | -2.97*** | -3.00*** | -3.41*** | -3.45*** | -3.50*** | -3.02*** |
| R-squared | 0.32 | 0.31 | 0.39 | 0.39 | 0.38 | 0.38 | 0.38 | 0.39 | 0.39 | 0.40 | 0.39 | 0.38 | 0.38 |
|  | **Charlson comorbidity index** | | | | | | | | | | | | |
| **VARIABLES** | **Treatment year 2000** | **Treatment year 2001** | **Treatment year 2002** | **Treatment year 2003** | **Treatment year 2004** | **Treatment year 2005** | **Treatment year 2006** | **Treatment year 2007** | **Treatment year 2008** | **Treatment year 2009** | **Treatment year 2010** | **Treatment year 2011** | **Treatment year 2012** |
| Treatment year | 0.06*** | 0.10*** | 0.15*** | 0.17*** | 0.19*** | 0.22*** | 0.23*** | 0.29*** | 0.30*** | 0.29*** | 0.30*** | 0.29*** | 0.30*** |
| Treatment group | -0.09*** | -0.11*** | -0.12*** | -0.13*** | -0.14*** | -0.16*** | -0.15*** | -0.13*** | -0.13*** | -0.12*** | -0.12*** | -0.11*** | -0.10*** |
| Interaction (DD estimate) | 0.03*** | 0.06*** | 0.10*** | 0.12*** | 0.12*** | 0.13*** | 0.13*** | 0.08*** | 0.09*** | 0.09*** | 0.07*** | 0.06*** | 0.02*** |
| R-squared | 0.12 | 0.13 | 0.14 | 0.15 | 0.15 | 0.17 | 0.17 | 0.17 | 0.16 | 0.16 | 0.16 | 0.16 | 0.16 |
| Observations | 1,347,732 | 1,372,878 | 1,427,275 | 1,466,762 | 1,477,191 | 1,495,694 | 1,523,483 | 1,510,378 | 1,510,716 | 1,507,550 | 1,502,141 | 1,508,812 | 1,519,431 |
| *** p<0.01, ** p<0.05, * p<0.1 | | | | | | | | | | | | | |

Table 11 Coefficients of interest from sensitivity analyses with varying end year in difference-in-difference analysis. Same included controls as table 2. Logistic regression, reported odds ratio.

|  | **Mortality within 30 days of admission** | | | |  |  | **Readmission** | | | |
| --- | --- | --- | --- | --- | --- | --- | --- | --- | --- | --- |
| **VARIABLES** | **Treatment year 2009** | **Treatment year 2010** | **Treatment year 2011** | **Treatment year 2012** |  | **VARIABLES** | **Treatment year 2009** | **Treatment year 2010** | **Treatment year 2011** | **Treatment year 2012** |
| Treatment year | 1.01 | 1.00 | 1.00 | 1.03** |  | Treatment year | 1.04*** | 1.13*** | 1.23*** | 1.11*** |
| Treatment group | 0.71*** | 0.71*** | 0.72*** | 0.72*** |  | Treatment group | 0.93*** | 0.93*** | 0.92*** | 0.93*** |
| Interaction (DD estimate) | 0.97 | 0.95*** | 0.93*** | 0.92*** |  | Interaction (DD estimate) | 0.97 | 0.92*** | 0.85*** | 0.86*** |
| Observations | 1,666,786 | 1,661,377 | 1,668,048 | 1,678,667 |  | Observations | 1,666,786 | 1,661,377 | 1,668,048 | 1,678,667 |
| Pseudo R-squared | 0.254 | 0.252 | 0.252 | 0.253 |  | Pseudo R-squared | 0.115 | 0.114 | 0.122 | 0.123 |
| *** p<0.01, ** p<0.05, * p<0.1 | | |  |  |  | *** p<0.01, ** p<0.05, * p<0.1 | | |  |  |
